# Self-competence increases the willingness to pay for social influence

**DOI:** 10.1038/s41598-020-74857-5

**Published:** 2020-10-20

**Authors:** Uri Hertz, Evangelia Tyropoulou, Cecilie Traberg, Bahador Bahrami

**Affiliations:** 1 Department of Cognitive Sciences, University of Haifa, 3498838 Haifa, Israel; 2 UCL Institute of Cognitive Neuroscience, University College London, London, WC1N 3AZ UK; 3 Department of Psychology, School of Biological Sciences, University of Cambridge, Cambridge, CB2 3EB UK; 4 Center for Adaptive Rationality, Max Planck Institute for Human Development, 14195 Berlin, Germany; 5 Faculty of Psychology and Educational Sciences, Ludwig Maximilian University, 80802 Munich, Germany; 6 Department of Psychology, Royal Holloway University of London, Egham, TW20 0EX Surrey UK

**Keywords:** Psychology, Human behaviour, Cognitive neuroscience, Social behaviour, Social neuroscience

## Abstract

Theoretical works in social psychology and neuroscientific evidence have proposed that social rewards have intrinsic value, suggesting that people place a high premium on the ability to influence others. To test this hypothesis, we asked whether, and under what conditions, people are willing to forgo monetary reward for the sake of influencing others’ decisions. In four experiments, online and lab-based participants competed with a rival for influence over a client. The majority of participants sacrificed some of their financial reward to increase their chance of being selected over their rival within the experiment. Willingness to pay was affected by the participant’s current level of influence and performance, as participants were most likely to pay to promote their competence after having given good advice that had been ignored by the client using a situation where monetary incentives fail to explain human motivations, our experiments highlight the intrinsic value of social influence.

## Introduction

Imagine hearing that a colleague at work is looking for some information about your area of expertise, and is asking your peers for advice. What would you be willing to do to make sure she turns to you? Would it matter if you think that your knowledge is going unnoticed? Would you be willing to strive for influence even if your peers are better equipped to answer? Humans seek the attention of their peers. People tell their friends that they are willing and able to solve their problems—and are affronted when their offer is ignored. Online, people carefully cultivate their social media presence in the pursuit of likes and followers. People are not satisfied by simply belonging to a group^1^; they spend a significant amount of time and cognitive effort enhancing their social status and influence^2–5^, comparing themselves to others^6–8^, and tracking others’ behaviour^9^. Indeed, seeking to increase one’s influence within a group is an important driver of human behaviour^2^. Recent findings from human neuroscience complement the behavioural and theoretical literature, and suggest that social rewards such as agreement with others^10^, inclusion in a group^11^, and elevated social status^12,13^ are processed in the brain similarly to primary and monetary rewards^14,15^. These observations suggest that people place a high premium on the ability to influence others and would spend material and social capital to gain social influence. However, this suggestion has not been tested empirically. We tested this intuition empirically, asking if, and under what conditions, people are willing to forgo money purely for the sake of influencing others’ decisions, even if that influence was conspicuously inconsequential.

While influence and social status can be achieved through coercion and dominance, another way to gain influence over others is by accumulating prestige and respect^16,17^. Group members assign high social status to prestigious individuals they perceive as having a unique ability or knowledge that they are willing to share for the benefit of others^18^. An important route for achieving social status, therefore, is to publicly demonstrate a willingness to put the group’s interests over one’s own^19^, for instance by donating money, sharing a skill, or teaching others^2^. Unadvertised virtues, however, cannot affect social status: Signalling one’s ability and willingness to help is crucial to climbing the social ladder^20^. Status-seeking individuals may use several strategies for self-promotion, including expressing confidence^21^ to exaggerate their virtues and engaging in self-promotion to distract from vices, cover up incompetence, or compensate for underwhelming performance^22^.

One manifestation of prestige and social influence is people voluntarily seeking and following an individual’s advice^16^. By giving advice, one offers privately held beliefs to a receiver, which may follow his advised course of action and hold the adviser accountable for its outcome. To obtain and maintain social influence one must communicate one’s privately held information carefully: Receivers are sensitive to the adviser’s accuracy, confidence and honesty^23^. They prefer confident advice^24^ and are willing to punish overconfident mistakes^25,26^. In a previous work using an advice-giving paradigm where participants competed with rival advisers for influence over a receiver, advisers expressed higher levels of confidence in their advice when their influence was low, thus taking a calculated risk^12^. When their influence over the receiver was high, they retreated to a more cautious policy, reducing their stated level of confidence in order to avoid future punishment if their advice turned out to be wrong. This finding was in line with a normative account of seeking and maintaining influence^27^ as well as with theories highlighting the fundamental role of social influence in human social behaviour^2,17,28^.

We took a social-cognitive approach to study how influence is sought and maintained, as well as the factors behind costly influence-seeking behaviour. We were interested primarily in characterising the factors affecting self-promotion on a trial-by-trial basis. As social influence is a manifestation of social status and prestige, social status theories suggest that a person’s level of influence relative to others within a group should affect their status-seeking behaviour^17^. In addition, theories of prestige formation suggest that self-promotion is linked with one’s own performance level and abilities, implying that performance level should affect costly influence-seeking behaviour^16,21^. We therefore hypothesised that participants will engage in costly self-promotion in order to increase their social influence, and that they will be most likely to pay a cost when their social influence is low while their performance level is high.

We used an advice-giving game in which two advisers competed for influence over a client^12^. We hypothesised that willingness to invest in self-promotion will be manifested in participants’ willingness to sacrifice some real-life financial reward to promote influence over virtual avatars in this game-like behavioural task. Each participant acted as an adviser over multiple trials and could use different strategies to increase and maintain their influence over the client: They could indicate their confidence level to the client, and, critically, they could forgo some of their real-life earnings from participating in the study in order to increase the probability of the client choosing them over their rival. The game allowed us to investigate the task-related factors affecting how participants used payments and confidence to strategically manage their influence.

## Results

In order to test our hypotheses that participants will use payments and pay for social influence and will do so more to promote their success than to gloss over failure, we conducted four experiments. Experiments 1 (N = 49) and 2 (N = 49) were carried out to examine willingness to pay for social influence after one’s success or failure is revealed, and previous level of influence over the client is known. Experiment 1 was conducted online and Experiment 2 in the lab. Experiment 3 (N = 53) was carried out to rule out the possibility that payments were merely affected by affordance or the participants’ eagerness to please the experimenter, and therefore we adapted the task such that similar payments affected the availability of information about influence, but not social influence directly. Experiment 4 (N = 50) was conducted to verify that self-promotion was associated with performance and not with uncertainty, and therefore we adapted the task such that participants could pay for influence *before* their success was revealed, but previous level of influence over the client is known.

**Figure 1:**
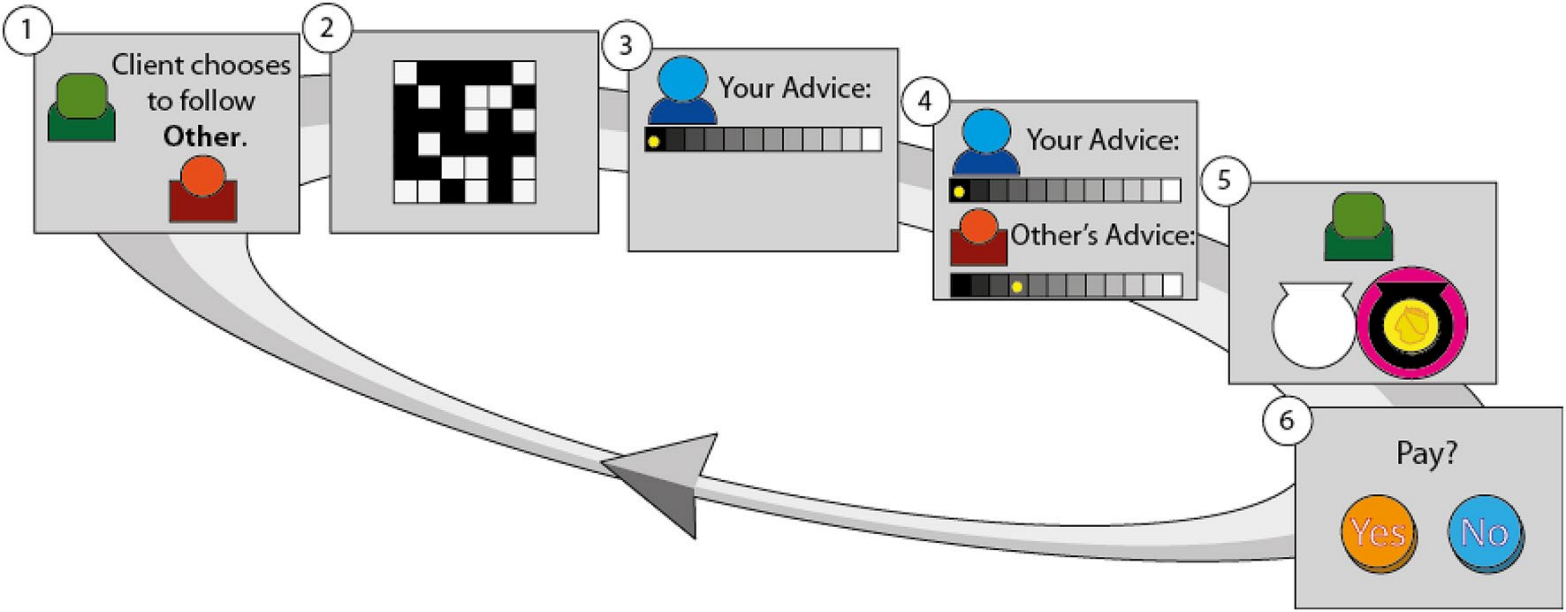
Schematic of one trial of the advice-giving task in Experiments 1 and 2. Participants played the role of an adviser competing with a rival adviser for influence over a client. Each trial consisted of six stages: (1) The client’s selected adviser was revealed, determining whose advice the client would follow in the upcoming trial (and consequently which adviser would be ignored). (2) The participant saw a grid of black and white squares whose ratio represented the probability of the coin being in the black urn. (3) The participant stated their advice on coin location using a 10-level confidence scale ranging from “definitely in the black urn” to “definitely in the white urn.” (4) Both advisers’ advice was displayed. (5) The content of the urn suggested by the selected adviser (magenta circle) was revealed. (6) The participants were asked if they were willing to pay to increase their influence on the client (thereby increasing the probability of being chosen by the client on the next trial). On the following trial the client could choose the same adviser or switch advisers according to the outcome of the recent trial and whether the participant paid to increase their influence. A working demonstration of the task is available at www.urihertz.net/AdviserDemoPay.

In all four experiments, participants played slightly different versions of an advising game^12^. In the basic advice-giving task two advisers competed for influence over a client’s attention (Fig. 1, see “Methods” for full details). We will describe the design of Experiments 1 and 2 here, and later how it was adapted in experiments 3 and 4. In experiments 1 and 2, on each trial, the client selected one of two advisers (the participant and a rival adviser) whose advice on which of two urns contained a hidden coin he will follow. At the beginning of each trial, the client chose the adviser to follow in that trial. The chosen adviser’s advice (given in a next step) would determine which urn would be opened. The client’s choice of adviser was displayed to the advisers (Fig. 1, panel 1), who were then shown the evidence (Fig. 1, panel 2). The advisers saw a grid of black and white squares for half a second. The ratio between the black and white squares indicated the probability of the reward location. Next (Fig. 1, panel 3), the advisers gave their advice on the location of the coin using a 10-level confidence scale. Both declarations were then shown to the two advisers and the client (Fig. 1, panel 4). Next, the urn indicated by the chosen adviser was opened; both the client and the advisers could see whether the urn contained the coin or not (Fig. 1, panel 5). Crucially, in the final step, the participant could forgo some of their real-world payment they would receive for taking part in the experiment in order to increase their influence over the client (if they did so, their probability of being selected by the client would increase) in the following trial (Fig. 1, panel 6). All participants played the role of an adviser, while the client and the rival adviser were played by computer algorithm (see “Methods” for a full description of the algorithms).

Participants’ overall payoff at the end of the experiment consisted of their flat-rate payment for participation minus the money they chose to forgo. Online participants were given a flat-rate of 4 USD for finishing the experiment, and their payments were deducted from an extra dollar—if they did not pay at all they received 5USD at the end of the experiment. Participants in the lab were given a flat rate of 8GBP for completing the task and their payments during the experiment were deducted from an additional 2GBP. Deduction was carried by randomly selecting 10 trials from the experiment, and deducting 10 cents for each payment trial in the online versions and 20 pennies per trial in the lab. Note that by paying on one trial the participant forgoes a relatively small sum of money, but the straightforward strategy for maximizing *payoffs* in our task was to never pay to influence the client.

This experimental design afforded an interactive social scenario which successfully engaged participants before^12^, allowing them to naturally assume the role of the adviser. In addition, this experimental setup was inspired by the contemporary concepts of social media (followers, influencers) and bestowed valuable ecological validity to our experiment. However, there are some notable limitations to this design. Our experiments resemble real life in that we often select our advisers (e.g. political leaders, investment adviser, physician) and then, we let them help us with their advice. However, quite often, we also are in a position to hear several advisers’ opinion before deciding who to follow. This latter aspect was not implemented in our experiment. In addition, in our experiments, participants did not engage in face-to-face interactions. This is, of course, also the case in many every day online and digital interactions, but still is a limitation of our experimental approach.

### Paying for social influence

Experiments 1 and 2 had the same structure but different cover stories and settings. Experiment 1 consisted of 130 trials carried out online, with a minimal cover story. Experiment 2 consisted of 190 trials and took place in a laboratory. Participants in Experiment 2 arrived in groups of three and were told that they were going to play the advice-giving game together. They did not know that they would each play the role of an adviser against virtual rivals and clients (see “Methods” for details).

**Figure 2:**
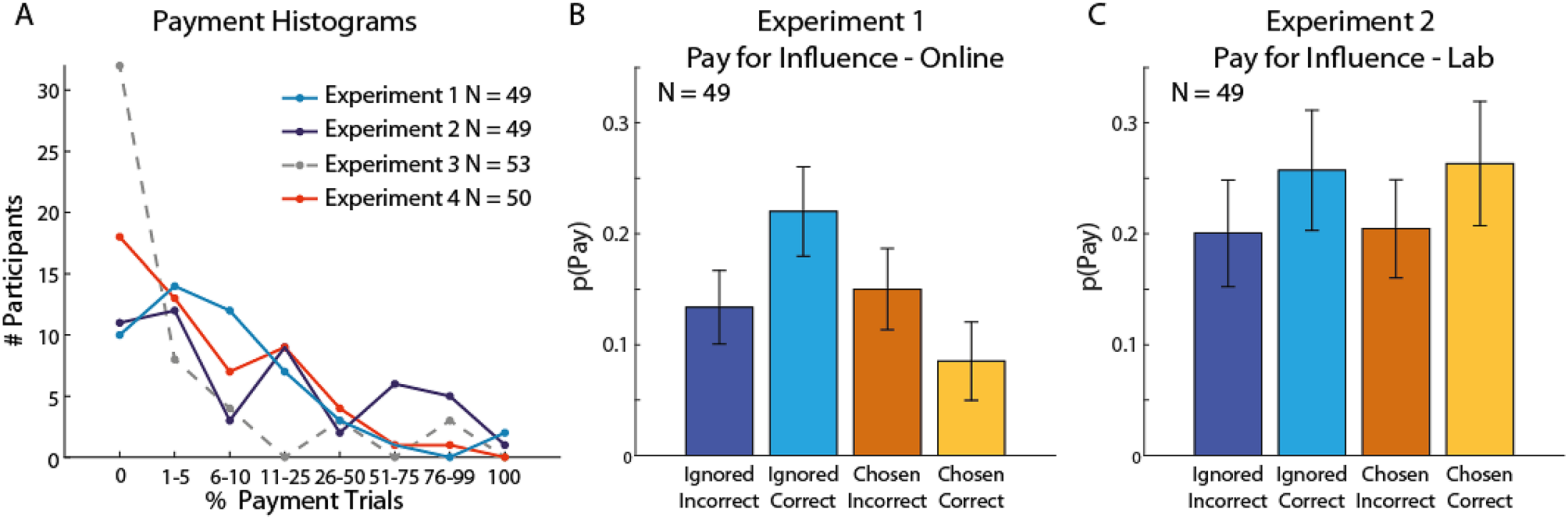
Paying for influence. (**A**) Histograms of the percentage of trials in which participants chose to pay, sacrificing their own endowment. In Experiments 1, 2, and 4 most participants chose to pay at least once, whereas in Experiment 3 most participants never chose to pay. Payment distribution was significantly different between Experiment 4 and all other experiments (p ≤ 0.014, two-sample Kolmogorov–Smirnov test). Payment distributions in experiments 1, 2, and 3 did not significantly differ from each other. (**B**, **C**) Strategic payment for influence. Participants paid for influence strategically: They were most likely to pay when the client had ignored them and their advice had been accurate in the current trial, i.e. just before the payment stage. Error bars represent SEM. Note that the plot serves only to illustrate the effects uncovered by the logistic regressions and should not be considered as a separate hypothesis testing.

We first examined the use of payment as a persuasive signal in Experiments 1 and 2. Consistent with our theoretical prediction that participants will be willing to sacrifice some of their endowment to increase their social influence^2,12^, most participants in Experiment 1 (39/49; Fig. 2A, light blue line), and Experiment 2 (38/49; Fig. 2A, black line) paid at least once for influence. While average payment rates were lower in Experiment 1 (12.1%; online) than in Experiment 2 (24.8%; laboratory), a direct comparison of the payment distributions between the two experiments using a two-sample Kolmogorov–Smirnov test did not show a significant difference (k = 0.24, p = 0.09).

### The effect of performance on willingness to pay for social influence

We used a trial-by-trial analysis to examine the factors affecting payments. We hypothesised that payment behaviour would be affected by participants’ current level of influence over the client—participants would be more likely to pay to increase their influence than to maintain influence. The participants’ influence level was revealed in the beginning of each trial (Fig. 1, stage 1), and could affect their decision to pay at the end of the trial (stage 6). We also hypothesized that participants’ willingness to pay would be affected by the accuracy of their recent advice (stage 5): if they wanted to promote their competence then they would be more likely to pay for influence after having given accurate advice; conversely, if they wanted to gloss over previous mistakes, they would be more likely to pay after inaccurate advice. Thus, our model tested the effect of two experimental variables: accuracy in recent advice (correct/incorrect) and influence over the client, captured by the client’s choice of adviser at the beginning of the trial (ignored/chosen).

We used a mixed-effect logistic regression model to examine the effect of experimental conditions on payment (see “Methods”). The model included advice accuracy, influence, and the interactions between the two, and two control variables, trial number and payment on previous trial. The model included group-level coefficients (i.e. fixed effects^29^), and intercept, influence and accuracy as individual-level coefficients (random effects) (pay ~ 1 + influence × accuracy + trial_num + previous_pay + (1 + influence + accuracy|id)) (Table 1). It showed a significant negative effect of advice accuracy (Beta ± SE: − 1.07 ± 0.18, t(6364) =  − 5.86, p < 0.0001), a significant negative effect of influence, i.e. more likely to pay after being ignored by the client (Beta ± SE: 0.56 ± 0.24, t(6364) = 2.37, p = 0.018) and a significant interaction effect (Beta ± SE: 1.38 ± 0.18, t(6364) = 7.78, p < 0.0001). These results indicate that participants were most likely to pay when they had previously been ignored by the client and their advice had been correct. They were less likely to pay for influence when their advice had been correct and they already had the client’s attention. These observations are illustrated in Fig. 2, panel B, which shows payment probabilities in four conditions resulting from crossing influence over the client (ignored/chosen) with advice accuracy (correct/incorrect). Rather than paying to gloss over their mistakes, participants used the payment to highlight their correct advice and advertise their competence.

**Table 1:** Summary statistics of the logistic regressions for Experiments 1 and 2.

Name	Estimate	SE	t (6364)	p	Lower CI	Upper CI
**Experiment 1** Pay ~ L(1 + Influence × Accuracy + Trial_num + Previous_Pay + (1 + Influence + Accuracy|id))						
Intercept	− 1.86	0.31	− 5.97	< 0.0001	− 2.47	− 1.25
Influence (Ignored)	0.56	0.24	2.37	0.018	0.1	1.03
Accuracy (Correct)	− 1.07	0.18	− 5.86	< 0.0001	− 1.43	− 0.7
Trial_num	− 0.005	0.0009	− 5.5	< 0.0001	− 0.006	− 0.003
Previous_Pay (Not)	0.78	0.089	8.76	< 0.0001	0.6	0.95
Influence (Ignored)* Accuracy (Correct)	1.38	0.18	7.78	< 0.0001	1.03	1.73

Name	Estimate	SE	t (9304)	p	Lower CI	Upper CI
**Experiment 2** Pay ~ L(1 + Influence × Accuracy + Trial_num + Previous_Pay + (1 + Influence + Accuracy|id))						
Intercept	− 1.82	0.3	− 6.1	< 0.0001	− 2.4	− 1.23
Influence (ignored)	0.19	0.23	0.82	0.41	− 0.26	0.64
Accuracy (correct)	0.03	0.25	0.12	0.9	− 0.46	0.53
Trial_num	− 0.003	0.00046	− 6.4	< 0.0001	− 0.004	− 0.002
Previous_Pay (Not)	0.73	0.06	12.17	< 0.0001	0.62	0.85
Influence (ignored)* accuracy (correct)	0.54	0.18	2.98	0.003	0.19	0.9

Participants in Experiment 2 performed the task in the laboratory and met other participants before carrying out the experiment in individual cubicles. These participants displayed a somewhat different pattern of strategic payment compared with the online cohort of Experiment 1. We used the same logistic regression model, and found that influence and accuracy did not affect payment behaviour significantly (Influence: Beta ± SE: 0.19 ± 0.23, t(9304) = 0.82, p = 0.41, Accuracy: Beta ± SE: 0.03 ± 0.25, t(9304) = 0.12, p = 0.9) (Table 1). However, the interaction between accuracy and influence was significant (Beta ± SE: 0.54 ± 0.18, t(9304) = 2.98, p = 0.003). Participants in the lab were most likely to pay for influence on trials when their influence was low, i.e. they were ignored by the client in the beginning of the trial, and their advice correct. To demonstrate these effects we averaged and plotted participants’ payments probabilities in the four experimental conditions according to selection by client and client reward (Fig. 2C). Lab-based participants were more likely to pay for influence after having given correct advice, and slightly more likely to pay when the client had also ignored them.

Despite their different settings, in both Experiments 1 and 2 participants’ behaviour was affected by the level of their influence over the client, i.e. whether they were ignored or chosen by the client at the beginning of the current trial, and the accuracy of their advice in the current trial, just before the payment stage. They were most likely to pay for influence on trials in which they were ignored by the client and their advice was correct, indicating that payment was aimed at promoting their abilities more than at glossing over their mistakes. The main difference between online and lab-based participants’ payment probabilities was observed when the client had chosen the participant and found the coin (see A+/C+ conditions in Fig. 2B,C). On these trials online participants were least likely to pay, but lab-based participants were as likely to pay as they were when they had predicted correctly but not been selected by the client. In addition, a base-rate of payments was observed in both experiments, with some likelihood of payment in all conditions.

### Ruling out alternative explanations

A criticism of Experiments 1 and 2 is that payment behaviour might be driven by mere affordances of the experiment or the participant’s willingness to comply with instructions. Similarly, one may argue that the financial stakes were so small that the paying participants may have been unconcerned about losing a bit of their pay. Because these concerns are fundamental to the interpretation of our findings, we designed a control experiment, Experiment 3, with similar affordance to Experiments 1 and 2, but with no social influence incentive. If participants in Experiments 1 and 2 had paid simply because the design afforded a non-specific chance to do so, merely to comply with instructions and please the experimenter or because the payment amount was insignificant, then Experiment 3 should produce in a similar pattern of probability of payments to that found in Experiments 1–2.

In this experiment the client’s choice of adviser was revealed on only half of the trials (random order). After the outcome had been displayed, participants were offered the opportunity to pay to increase the probability of *observing* the client’s choice of adviser in the following trial, and therefore view their own influence level. However, payment did not affect the probability of being chosen on the next trial, i.e. it did not affect their level of social influence. This design featured the same payment affordance as Experiments 1 and 2, but without payment leading to changes in the participant’s influence over the client. In addition, previous studies have shown that information about monetary gains and losses may incentivise participants’ payment behaviour^30^, so one can still predict that information about social influence will merit sacrifice of some monetary compensation. This experimental design therefore tested whether information about social influence merits forgoing endowment. If participants were not willing to forgo some of their payment for taking part in this experiment, we would be able to conclude that the payment behaviour in Experiments 1 and 2 was not the mere consequence of an available affordance, and that changes to social influence were the key incentive to lead participants to spend some of their hard-earned money.

We recruited a new cohort of participants online to participate in Experiment 3. The payment structure was the same as in experiments 1 and 2. Participants received a flat-rate of 4USD for participation, and their payments in the experiment were deducted from an additional 1USD. The amount of deduction was determined by ten trials, randomly selected for each participant, and 10 cents were deducted for every trial in which the participant chose to pay. A participant that did not pay on any trial therefore received 5USD. The majority of participants in this experiment did not pay for influence information even once (32/50). The distribution of payment rates of Experiment 3 (Fig. 2A) was significantly different from both Experiments 1 and 2 (two-tailed Kolmogorov–Smirnov tests: Experiment 3 vs Experiment 1: k = 0.45, p < 0.0001, Experiment 3 vs Experiment 2: k = 0.41, p = 0.0002). This result indicated that simply presenting the option to pay was not enough to drive the payment behaviour observed in Experiments 1 and 2; therefore, a nonspecific obligation to follow instructions could not explain the payment probabilities. In this sense, the distribution of payment in Experiment 3 serves as a well-controlled baseline for what may be called unmotivated, random payment behaviour.

### Self-promotion and prospective success

**Figure 3:**
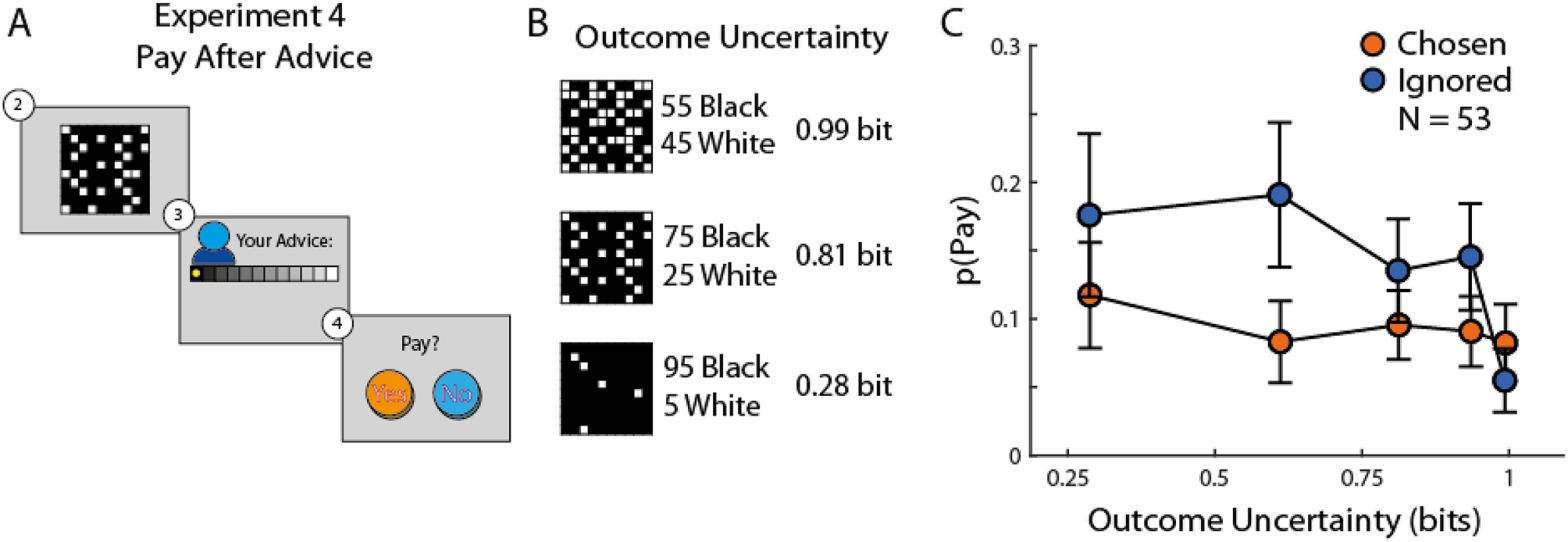
Payment relation to evidence uncertainty. (**A**) In Experiment 4 the option to pay was shown after the participant and rival had given their advice but before the coin location was revealed. Note that the rest of the stages were the same as experiment 1 (Fig. 1). (**B**) We formalised outcome uncertainty using the grid information entropy. (**C**) Payments in Experiment 4 were related to outcome uncertainty, as revealed by mixed-effect logistic regressions. When participants were ignored, they were more willing to pay when uncertainty was low; this demonstrates that payment was aimed at promoting one’s ability when the participants’ prospective success is high. Error bars indicate standard error of the means (SEM).

Experiments 1 and 2 showed that participants were willing to pay for influence after giving accurate advice, and less so after giving advice that turned out to be wrong, suggesting that payment was made to promote one’s competence. To expand on this suggestion, we designed Experiment 4 to test the relation between self-promotion and prospective performance, i.e. the probability of being correct before outcome is revealed. We modified the paradigm such that the participant was offered the opportunity to pay for influence over the client immediately after giving advice and just before the rival’s advice and the outcome were revealed (Fig. 3A). Payment structure for this experiment was the same as in Experiments 1 and 3. In this case participants’ payment was likely to be influenced by the outcome uncertainty, represented by the ratio of black and white squares in the evidence grid, and not just by the outcome of the lottery and the accuracy of their advice (Fig. 3B). Participants could predict their advice accuracy based on the evidence uncertainty, with low uncertainty being more likely to lead to choosing the correct urn. Participants could therefore choose to pay when uncertainty was high in order to compensate for the possibility of being wrong, or when uncertainty was low in order to promote their high chances of being correct. In addition, just like in previous experiments, their payment behaviour was expected to be affected by their current level of influence—were they chosen or ignored by the client in the beginning of the trial. Our results from Experiments 1 and 2 are in line with the latter alternative and predict that payment should increase when uncertainty decreases, i.e. payment will be associated with prospective success.

We recruited a new cohort of online participants for Experiment 4. The results replicated our main finding: The majority of participants used payments to increase their influence in at least in one trial (35/53; Fig. 2A), and the payments distribution in Experiment 4 was significantly different from the baseline distribution in Experiment 3 (Experiment 4 vs Experiment 3: k = 0.3, p = 0.014). The average payment rate was 18.5%, and the distribution of payments was similar to the distribution in Experiment 1 (Experiment 4 vs Experiment 1: k = 0.22, p = 0.14) and Experiment 2 (Experiment 4 vs Experiment 2: k = 0.23, p = 0.12).

To test our prediction that payments will be affected by prospective success rather than to compensate upcoming failure, we examined the relation between evidence uncertainty and payment. We used a mixed-effect logistic regression model that included the influence factor (ignored/selected), the outcome uncertainty factor, and the interaction of the two as fixed effects. Outcome uncertainty was captured by using the evidence information entropy in units of information bits, according to the formula:

1 $$S = - \left( {p_{black} \cdot \log_{2} \left( {p_{black} } \right) + p_{white} \cdot \log_{2} \left( {p_{white} } \right)} \right),$$

where *p*_*black*_ is the proportion of black squares in the evidence grid (Fig. 3B) and *p*_*black*_ + *p*_*white*_ = 1. When the grid contains 50 black squares and 50 white squares, indicating high uncertainty about the outcome (the coin’s location), evidence entropy is high and the participant needs 1 bit of information to completely disambiguate the outcome. On the other hand, when the grid has 95 black squares, entropy is much lower and the participant needs no more than 0.28 bits of information.

We used outcome uncertainty as an ordinal variable in our mixed-effects logistic regression, and the current level of influence (ignored/chosen) as a nominal fixed effect. We also included two control variables, the trial number and the payment on previous trail. As before, the model included group-level coefficients (fixed effects), and intercept, influence and uncertainty as individual-level coefficients (random effects) (pay ~ 1 + influence × uncertainty + trial_num + previous_pay + (1 + influence + uncertainty|id)) (Table 2). We found a significant influence effect (Beta ± SE: 1.73 ± 0.5, t(3704) = 3.48, p = 0.0005), indicating a higher probability to pay for influence when ignored by the client at the beginning of the trial. The uncertainty effect was not significant (Beta ± SE: 0.82 ± 0.47, t(3704) = 1.73, p = 0.08). However, we found a significant interaction effect (Beta ± SE: − 1.23 ± 0.54, t(3704) = − 2.26, p = 0.024), indicating that the likelihood that a participant would pay for influence increased as the uncertainty about the outcome decreased, and the participant’s influence level was low.

**Table 2:** Summary statistics of the logistic regression for Experiment 4.

Name	Estimate	SE	t (3704)	p	Lower CI	Upper CI
**Experiment 4** Pay ~ L(1 + Influence × Uncertainty + Trial_num + Previous_Pay + (1 + Influence + Uncertainty |id))						
Intercept	− 3.04	0.5	− 6.11	< 0.0001	− 4.01	− 2.06
Influence (ignored)	1.73	0.5	3.48	0.0005	0.75	2.7
Uncertainty	0.82	0.47	1.73	0.08	− 0.11	1.75
Trial_num	− 0.0043	0.002	− 2.21	0.027	− 0.008	− 0.0005
Previous_Pay (Not)	0.37	0.1	3.68	0.00023	0.17	0.57
Influence (ignored)* uncertainty	− 1.23	0.54	− 2.26	0.024	− 2.37	− 0.16

To directly demonstrate the effect of prospective success on payment, we calculated the rate of payments in 10 experimental conditions resulting from combining the five outcome uncertainty levels (0.28–0.99 bit) and two influence levels (ignored/selected; Fig. 3C). We found that the likelihood of payment decreased with outcome uncertainty only when the participant was ignored by the client. These results indicate that payment was not used to gloss-over a high probability of mistake, but rather to promote one’s ability when prospective success is high. This result is in line with the increased likelihood of payment after giving accurate advice in Experiments 1 and 2. In Experiments 1, 2, and 4, the use of payment as a mean of self-promotion was linked to the participants’ advice accuracy.

Finally, we examined the use of advice confidence as a persuasive signal. Our previous findings using the advice-giving game showed that participants reported their advice confidence strategically to gain and maintain influence over the client^12^. Replicating our previous work, participants in Experiments 1, 2, and 4 expressed higher confidence in their advice when the client ignored them and reduced their reported level of confidence when the client chose them, in line with a normative prescription of social-influence-seeking strategy^27^ (see Supplementary Information).

## Discussion

In four experiments we examined people’s willingness to incur small real-world monetary losses in order to increase their influence over a client. We found that the majority of our participants, both in the laboratory and online, were willing to pay at least on one trial, to increase their influence. Payment for influence was affected by the participants’ trial-by-trial level of influence over the client and their competence: Likelihood of payment increased if the participants were ignored by the client and their recent advice turned out to be correct. This pattern reoccurred even when participants had to rely only on their likelihood of being successful before making the payment, where they paid for influence when they were ignored by the client and the uncertainty about the outcome was low. Payment was not the only means used to increase the participants’ influence: Participants also used their confidence in their advice as a persuasive signal. Participants’ advice confidence was affected by their level of influence but not by their performance level, as participants expressed more confidence when their influence was low and moderated it when their influence was high.

A pervasive tenet of studies in decision making and behavioural economics is that participants should be properly incentivised, usually by a promise of increased monetary reward; otherwise the experimenter cannot be sure that participants would be adequately and appropriately motivated to carry out their task. Here we have shown that monetary incentives fail to capture the entire extent of human motivations—social influence is a strong incentive, even when it entails monetary loss. Our findings provide direct evidence of both the intrinsic value of social influence and the importance of self-competence in social behaviour. These findings support the theory that social outcomes, and not just material or monetary outcomes, may be fundamental motivations for behaviour^2,17,31,32^.

But what drove our participants to incur real-world cost merely to increase the likelihood of being selected by an avatar? First, it should be noted that the amount at stake on each trial, in every payment decision, was not very high: if this kind of trial is selected at the end of the experiment, which had a chance of 10/130 online, the participant will lose 10 cents. With many participants paying very few times in our experiments, the amount they risked was not very high. Nevertheless, the obvious strategy to maximize one’s reward was to never pay, and this strategy was adopted overwhelmingly by participants in control Experiment 3, when payment did not affect influence.

Another reason for choosing to pay for influence in some of the trials has to do with the value of influence and appraisal by others. Baumeister and Leary suggested that belonging to a group is a fundamental motivation for behaviour, and indeed people readily form strong interpersonal connections and are willing to exert effort to maintain their social connections^33^. It has also been suggested that gaining and maintaining social status within a group is yet another fundamental motivation for behaviour^2^. The negative effect of social exclusion, or ostracism, has been demonstrated in multiple studies. Some of the most convincing results have come from the study of social exclusion in ‘cyberball’ paradigm^34,35^. In this computer-based game, the participant and two avatars throw a ball to each other and social exclusion is triggered when the avatars avoid throwing the ball to the participant. Participants in this task report decreased mood and satisfaction and increased distressed when ostracised^36^. Relevant to our findings, participants were affected by ostracism even when they were told that they were playing against a computer that was pre-programmed to ignore them^36^, and even when being ignored resulted in increased monetary rewards^37^. Social incentives may therefore be triggered even in virtual environments, where the actual identity of the other players is unknown, and even when they are uncoupled from monetary compensation^38^. Indeed, participants in a previous version of the current advice-giving task displayed similar pattern of responses when playing with virtual agents and when playing with other participants^12^, with and without monetary incentives. In our case, gaining influence, i.e. being selected by the client, could serve as an incentive strong enough to incur some monetary loss under some conditions.

These social motivations are supported by neuroimaging studies in social neuroscience^4,12,39–42^, which showed that social rewards such as reputation and increased social status were strong drivers of the brain’s reward system, the same system that responds to primary rewards such as food and secondary ones such as money^15^. Other, more indirect social rewards (e.g., getting confirmation for one’s opinions from others) also activate this valuation system, while misalignment with the group leads to decreased activity of the reward network^10,43,44^. Our results go beyond observational reports of brain activity and provide direct support for social influence and acceptance having an intrinsic value. Our results suggest that, like social identity^32^, social status should be factored into the economic utility function to explain human decisions and should be considered when evaluating experimental incentive structure.

An important finding in our study is that influence was not purchased randomly; instead, it was closely linked to task-related factors. Participants were more likely to pay when their influence over the client was low, and less likely to pay when their influence was high. This is consistent with our hypothesis that payments would be made to increase social influence, and is in line with findings showing that status-minded individuals tend to be more generous^45^. Participants also paid for influence more often when their advice was accurate or likely to be accurate—that is, when they had something positive to show off. Similarly, previous results using the advice-giving task showed that participants registered higher levels of confidence in their advice to gain influence when they perceived themselves as more accurate than their rival but had been ignored by the client^12^. The importance of signalling one’s competence in the pursuit of status highlights an important aspect of social status. As prestigious individuals possess a unique knowledge, ability, or skill they can share with the group^16,18^, the first step in seeking prestige may be to signal the possession of such a capability, even to oneself^46^. Anderson et al. increased participants’ sense of capability by providing them with fictitious positive feedback about their performance, leading the participants to appear more confident in a dyadic interaction and enjoy a boost to their social status. The opposite of this effect has also been observed: One is less likely to engage in self-promotion when one feels incapable (i.e., when one is not sincere in promoting their competence, or done to cover up a lack of competence). These findings supported our hypothesis that social status seeking is linked to signalling competence. If people are just as likely to pursue status regardless of their capabilities, self-promotion will not be an efficient strategy, since too many people will engage in hollow self-promotion. Of course, this does not apply to people with misguided perception of their ability, which may be willing to promote their prospective success with high conviction, even though in truth they are likely to be wrong. With no direct incentive to tell the truth, people might tell the truth, but could not do so in a verifiable way—a situation known in economics as “cheap talk”^47^. However, the pattern of behaviour we observed, by which people were more engaged in self-promotion when their performance level was high may prevent cheap talk situations in real life and could promote confidence signals as informative and reliable.

In addition to paying for influence, our participants also reported higher levels of confidence in their advice when they had been ignored by the client. This behaviour is consistent with a previous study in which participants did not have the option to pay for influence^12^, and in line with a normative prediction of strategic report of confidence aimed to increase their probability of being selected by the client in future trials^27^. This pattern is similar to the results we observed of an increased likelihood of paying for influence on trials when advisers had been ignored by the client, since both payment and confidence served as tools for self-promotion. This pattern is also in line with theoretical accounts of the role of confidence and information disclosure called cheap talk^47^. Cheap talk refers to situations of information asymmetry between a seller (or multiple sellers) and a client, and examines the best strategy for information disclosure for sellers to secure a buy from the client. These works indicate that in many scenarios it is optimal for an adviser to not disclose all her private information^48^, for example by catering their advice to the preferences of the client^49^. The exaggerated advice confidence given to gain influence is in line with such theoretical accounts. However, our participants did not exaggerate their advice too much (or as much as some models predict^27^), and preferred to honestly promote their competence using payments. It may indicate that they are also aware of advisee’s sensitivity to advisers’ honesty^23^, accounting for their divergence from more extreme self-promotion strategies.

In our study participants played the role of advisers competing against a virtual adviser to win the attention of a virtual client, providing greater control and consistency across participants. In an earlier study we compared behaviour in the advice-giving task in a similar setting with virtual agents to a version in which 3 human participants played these roles in a live, interactive version of the task^12^. The results showing that advisers behaved similarly in both cases supported the idea that the algorithms implemented in our current study could adequately capture the key elements of the social interaction. The cover stories used in our current study varied slightly between online and lab-based versions of the task (see Supplementary Information). Lab-based participants arrived in groups of three and were told they would be playing against each other, but then played independently in isolated cubicles. They were told that payments would be used for an advertising campaign to increase the likelihood of the client choosing them. This was done to make it clear that their payment would not go directly to another participant. Online participants were not given any information about the identity of the other participants and were told simply that payments would increase the probability of being chosen by the client. These minimal instructions were enough to trigger payment behaviour online.

There was a good level of agreement between the distribution of payments from the online and lab-based experiments, even though the details of the cover story provided to the participants differed. Lab-based and online participants paid for influence and did so according to their level of influence and their probability of being correct. These findings support the notion that online platforms, such as Amazon Mechanical Turk, are valid platforms for social-cognition experiments^50,51^. In addition, participants in the online experiment were older and came from a wider range of ages compared to lab-based participants. Combining lab-based and online based populations was useful for diversifying our cohort, as well as for demonstrating the generality and robustness of our main findings.

Payment strategies derived from the analysis of the pattern of payment behaviour online (Experiment 1) and in the laboratory (Experiment 2) shared some key common elements and differed in others. Lab-based participants paid more on average, but payment distribution did not differ between online and lab-based experiments. Payment strategies derived from analysing the pattern of payment behaviour also shared some, but not all, features. The biggest divergence between online and lab-based participants in Experiments 1 and 2 was in payment after having been accurate when selected by the client (Fig. 2): Here, lab-based participants were more likely to pay for influence than were online participants (Fig. 2). It is possible that in the more convincing social setting of the laboratory, participants used payment to signal competence more consistently and emphatically to maintain their influence and status^45^.

## Conclusion

To conclude, we have demonstrated that in social interactive setting, gaining influence over others’ behaviour can override the motivation to maximize monetary benefit. Participants were willing to forgo financial reward in order to gain influence. They promoted themselves by paying for influence and claiming to have higher levels of confidence, paying more and expressing more confidence when ignored by the client. Participants were selective in their use of self-promotion and were more likely to pay for influence when their advice had been or was likely to be accurate. Our results support the social psychological theories that posit an intrinsic value for social influence, and highlight the role of self-competence in the deployment of costly self-promotion signals—an important aspect of reputation building and prestige as sources of social status.

## Methods

### Participants

We recruited four cohorts of participants for this study. All participants provided informed consent and received monetary compensation. All groups were approved by the UCL Research Ethics Committee, and all experiments were performed in accordance with relevant guidelines and regulations. In a previous study using the advice-giving game^12^ we found an effect size of 0.5 for our main result. While that study was aimed at evaluating other aspects of the advice-giving task, we used this effect size for power calculation, and found that N = 44 should give significant results with a power of 0.9. For Experiment 1, carried out online, we recruited 49 participants (26 male, aged 31 ± 8 [mean ± std]; 23 female, aged 38 ± 11) using Amazon Mechanical Turk. For Experiment 2, a lab-based study, we recruited 49 participants (18 male, aged 24 ± 4; 31 female, aged 24 ± 3). For Experiment 3, carried out online, we recruited 50 participants (28 male, aged 34 ± 10; 22 female, aged 36 ± 7) using Amazon Mechanical Turk. For Experiment 4, carried out online, we recruited 53 participants (28 male, aged 35 ± 10; 25 female, aged 36 ± 9) using Amazon Mechanical Turk.

### Advice-giving task

We employed a variant of an earlier advice-giving game^12^ (Fig. 1). In this task the participant played the role of adviser, competing with a rival adviser for influence over a client. On each trial, the client selected one of two advisers (the participant and a rival adviser) whose advice on which of two urns contained a hidden coin he will follow. At the beginning of each trial the client’s choice of adviser was displayed to the advisers (Fig. 1, panel 1), who were then shown the evidence (Fig. 1, panel 2). The advisers saw a grid of black and white squares for half a second. The ratio between the black and white squares indicated the probability of the reward location. Next (Fig. 1, panel 3), both advisers gave their advice on the location of the coin using a 10-level confidence scale. Both declarations were then shown to the two advisers and the client (Fig. 1, panel 4). Next, the urn indicated by the chosen adviser was opened; both the client and the advisers could see whether the urn contained the coin or not (Fig. 1, panel 5).

Experiments 1 and 2 included an additional final step in which the participant could forgo some of the real-world payment they would receive for taking part in the experiment in order to increase the probability of being selected by the client in the following trial (Fig. 1, panel 6). Payment on a trial increased the likelihood of being selected by increasing the client’s influence weight assigned for the participant (see Supplementary methods). In Experiment 4 the payment stage was moved to be immediately after giving advice, and before observing the other adviser’s advice and the outcome (Fig. 3A). In Experiment 3 participants did not pay for influence, but to increase the likelihood of observing the client’s choice of adviser and their influence level on the next trial. In this experiment this probability was set to 0.5, and increased to 0.8 after payment trials.

More details about the experimental procedure are included in the Supplementary methods.

### Procedure

Participants in the online cohorts (Experiments 1, 3, and 4) completed the advice-giving game using their own personal computers. No cover story was provided regarding the identity of the rival adviser and client. The instructions said that participants could pay the client to increase the probability of being selected. Choosing to pay for influence could jeopardize up to $1 of their total $5 monetary compensation, which they received at the end of the experiment. Instructions clearly stated that at the end of the experiment, 10 trials would be randomly selected and for each payment decision sampled by this selection, 10 cents would be deducted from their sum total. Experiment 1 consisted of 130 trials; Experiments 3 and 4 each consisted of 70 trials.

In Experiment 2, lab-based participants were invited to the laboratory in groups of three. They were told that they would play an advice-giving game together, and that the roles of two advisers and a client would be assigned randomly by the computer program at the beginning of the experiment. The participants then were seated individually in isolated cubicles. Unbeknownst to the participants, each participant was assigned the role of adviser while the behaviour of rival adviser and client were controlled by the experimental code. To avoid the implication that more money would be paid to the participant assigned to the client role, participant’s payments were described as funding an advertising campaign that would increase the chance of being selected in the following trial. For lab-based participants, choosing to pay for influence could jeopardize up to £2 of their total £10 monetary compensation. Experiment 2 consisted of 190 trials.

### Model-fitting procedure

To examine payment behaviour, we used mixed-effects logistic regression models. Participants’ trial-by-trial payment served as the dependent variable, and the trial-by-trial influence over the client (ignored/chosen), advice accuracy (incorrect/correct), and outcome uncertainty in bits (Eq. 1) were used as dependent variables of interest, and trial number and payment on previous trial were used as control variables. Our models are multilevel models^52^, and include group-level coefficients, referred to in the text as fixed effects^29^, and individual-level coefficients including the intercept, influence, uncertainty and accuracy, referred to in the text as random effects^29^. All models were fitted using Matlab R2018b Statistics Toolbox (MathWorks Inc.).

## Supplementary information

Supplementary Information.

## Data Availability

The behavioural data and scripts supporting the findings of this study are available from figshare (https://figshare.com/s/fada1567a5acda46bd1e).
