# Supplementary material for: Self-competence increases the willingness to pay for social influence
Source: Sci Rep. 2020 Oct 20;10:17813. doi: 10.1038/s41598-020-74857-5 (PMC7576769; doi:10.1038/s41598-020-74857-5)
Supplement: Supplementary file 1 — Supplementary Information. [file 41598_2020_74857_MOESM1_ESM.docx]

**Self-Competence Increases the Willingness to Pay for Social Influence**

Uri Hertz^1^*, Evangelia Tyropoulou^2^, Cecilie Olesen^3^, Bahador Bahrami^4,5,6^

^1^ Department of Cognitive Sciences, University of Haifa, Haifa, 3498838, Israel

^2^ UCL Institute of Cognitive Neuroscience, University College London, London, WC1N 3AZ, United Kingdom

^3^ Department of Psychology, School of Biological Sciences, University of Cambridge, Cambridge, CB2 3EB, United Kingdom

^4^ Center for Adaptive Rationality, Max Planck Institute for Human Development, Berlin, 14195, Germany

^5^ Faculty of Psychology and Educational Sciences, Ludwig Maximilian University, Munich, 80802, Germany

^6^ Department of Psychology, Royal Holloway University of London, Egham, Surrey, TW20 0EX, United Kingdom

**Supplementary Materials**

**Methods - Task**

Our experiment design followed the design in Hertz et al. 2017 ^1^. In all experiments participants played the role of an adviser, while the client and rival adviser were played by computer algorithm. The rival adviser’s advice was calculated on each trial according to the probability of the coin being in the black urn (0–1), plus noise ( $\sim N\left(0,0.08\right)$), to range between [5W 5B], just like the participants’ advice. In each trial, after the outcome was revealed the prognostic value of the advice from the participant and their rival was calculated by multiplying the confidence level (1–5) by accuracy (indicating the correct coin location, i.e., W for white or B for Black urn; 1 = correct, -1 = incorrect).

The client’s choice of adviser was determined by assigning an influence weight to each adviser, updating the weights after each outcome, then choosing the adviser with the higher weight for the next trial. The weights summed to 10 and were set at 5 for each adviser at the start of the experiment. To update the client influence weights, we used the prognostic value of advice (PA) for each adviser. We first multiplied the advisers’ absolute confidence (1–5) by accuracy (1 correct, -1 wrong), and added 5 to make it range between 1 and 10. We used the notation $PA_{P}$ to refer to prognostic value of the participant’s advice and $PA_{O}$ for the prognostic value of the rival’s advice. The weights were updated after each trial according to the last trial’s prognostic values, following a rule similar to the one used by Bayarri and DeGroot ^2^:

[2] $w_{P}\left(t+1\right)=10\cdot\frac{w_{P}\left(t\right)\cdot PA_{P}\left(t\right)^{2}}{w_{P}\left(t\right)\cdot PA_{P}\left(t\right)^{2}+w_{O}\left(t\right)\cdot PA_{O}\left(t\right)^{2}}$,

where $w_{P}$ is the influence weight assigned to the participant. When both advisers give the same advice the influence weights remain the same.

In Experiments 1, 2, and 4, participants could pay to increase their influence—that is, the probability of being selected by the client in the next trial. In Experiments 1 and 2 this opportunity was presented at the end of the trial, after the outcome had been revealed (Figure 1), and in Experiment 4 it was presented after the advisers had given their advice but before the rival adviser’s advice and outcome were revealed. After the influence weights were updated, we added 3 to a participant’s influence weight in trials in which they chose to pay for influence. To increase the probability of the client switching between advisers, maximum and minimum bounds were set for influence weights at 8 and 2, respectively.

In Experiment 3 we used a different version of the advice-giving task in which payment affected the probability of the participant being able to see the client’s choice of adviser, rather than affecting influence over the client. In this version, the client’s choice of adviser had a 0.5 probability of being shown to the participant. On trials in which the client’s choice was not presented, a message that client selection was unknown was displayed instead. The participants could pay after the outcome stage to increase the probability of observing the client’s choice in the following trial and see their own influence level. If they chose to pay, the probability of observing the client’s choice increased to 0.8.

In accordance with our earlier implementation of the advice-giving task ^1^, we selectively manipulated the participant’s and the rival adviser’s advice quality on some trials. This was done to avoid cases in which one adviser is always picked by the client while the other is always ignored. Therefore, in restricted periods of trials, we introduced some noise to one of the two advisers’ evidence such that the ratio of black and white squares in the grid became a poor predictor of the coin’s location. The procedure was as follows: If in a specific trial the probability of the coin being in the black urn was 0.75, the grid would normally include 75 black and 25 white squares. On a noisy trial with similar probability of 0.75, this composition would change to 55 black squares and 45 white squares, a reduction in contrast by 20 squares. In all noisy trials contrasts were reduced by 20 squares in a similar fashion. The procedure ensured that one adviser’s advice accuracy was systematically inferior to the other’s for a number of consecutive trials, thus increasing the probability that the virtual client would shift to selecting the other adviser. This procedure was implemented for 10 trials every 30 trials (10 noisy trials followed by 20 non-noisy trials) and favoured the participant and the rival adviser alternately.

To ensure that manipulation of evidence quality was not the sole driver of changes in accuracy and difficulty throughout the task, the order of the evidence displayed to participants in each trial (the ratio between black and white squares in the grid) was randomised for each participant. In addition, the coin location was randomly generated in each trial according to the evidence (evidence only implied the probability of the coin location). This meant that each participant experienced individual periods of high/low accuracy, which were not related to the timing of the evidence quality manipulation.

**Confidence as a persuasive signal**

We examined the use of advice confidence as a persuasive signal^1^. To assess the strategic use of advice confidence, we examined the trial-by-trial deviance of advice confidence from probabilistic evidence. If the adviser is strictly committed to communicating the information they are given, then confidence exactly matches the ratio of black to white squares in the evidence grid (Figure 1, main text). Advice confidence would deviate if the confidence is higher (positive deviance) or lower (negative deviance) than the probability indicated by the evidence. We therefore examined whether advice deviance in a trial was affected by influence over the client (ignored/chosen) in Experiments 1, 2, and 4 using multi-level regression analysis ^3^ (Table S1). In all three experiments, we found a significant effect of influence over the client: Advisers increased their advice deviance when they were ignored by the client compared to when they were chosen by the client (Table S1). Analysis of advice confidence revealed that, in line with previous results, participants used their advice confidence as a persuasive signal, increasing it when seeking influence and becoming more conservative once influence was gained.

**References**

1. Hertz, U. *et al.* Neural computations underpinning the strategic management of influence in advice giving. *Nat. Commun.* **8**, 2191 (2017).

2. Bayarri, M. J. & DeGroot, M. H. Optimal reporting of predictions. *J. Am. Stat. Assoc.* **84**, 214–222 (1989).

3. West, B. T., Welch, K. B. & Galecki, A. T. *Linear mixed models: a practical guide using statistical software*. (CRC Press, 2014).


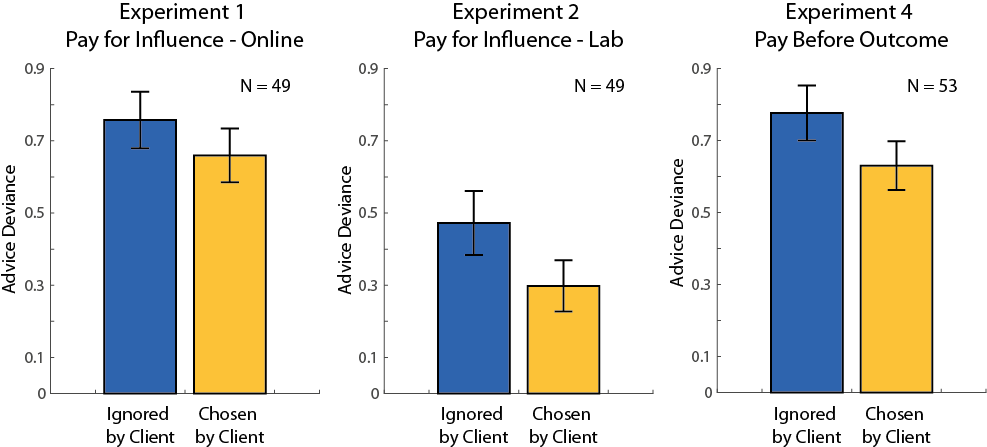


*Figure S1*. Influence effect on advice deviance. We measured participants’ advice deviance, that is, the deviance of their confidence in their advice from probabilistic uncertainty presented in each trial. Following our mixed-effects model, we examined advice deviance in two conditions according to selection by client (ignored/selected). The plots demonstrate the main effect of influence: Participants were expressed higher confidence in their advice when they were ignored by the client than when they were chosen, in all three experiments. (Error bars represent SEM.)

| **Experiment 1**  AdviceConfidence~1+ Influence + Trial_num + Previous_Pay + (1|id)) | | | | | | |
| --- | --- | --- | --- | --- | --- | --- |
| Name | Estimate | SE | t (6366) | p | Lower CI | Upper CI |
| Intercept | 0.63 | 0.077 | 8.2 | >0.0001 | 0.48 | 0.79 |
| Influence (Ignored) | 0.073 | 0.027 | 2.69 | 0.007 | 0.02 | 0.12 |
| Trial_num | -0.00023 | 0.0003 | -0.73 | 0.46 | -0.0008 | 0.00037 |
| Previous_Pay (Not) | 0.242 | 0.047 | 5.18 | >0.0001 | 0.15 | 0.33 |
| **Experiment 2**  AdviceConfidence~1+ Influence + Trial_num + Previous_Pay + (1|id)) | | | | | | |
| Name | Estimate | SE | t (9306) | p | Lower CI | Upper CI |
| Intercept | 0.47 | 0.072 | 6.49 | >0.0001 | 0.32 | 0.61 |
| Influence (Ignored) | 0.15 | 0.022 | 6.7 | >0.0001 | 0.107 | 0.19 |
| Trial_num | -0.002 | 0.00016 | -13.35 | >0.0001 | -0.0024 | -0.0018 |
| Previous_Pay (Not) | 0.062 | 0.031 | 2.001 | 0.045 | 0.0012 | 0.12 |
| **Experiment 4**  AdviceConfidence~1+ Influence + Trial_num + Previous_Pay + (1|id)) | | | | | | |
| Name | Estimate | SE | t (3706) | p | Lower CI | Upper CI |
| Intercept | 0.55 | 0.069 | 8.04 | >0.0001 | 0.42 | 0.69 |
| Influence (Ignored) | 0.094 | 0.034 | 2.75 | 0.0058 | 0.027 | 0.16 |
| Trial_num | 0.0017 | 0.0007 | 2.35 | 0.019 | 0.00028 | 0.0031 |
| Previous_Pay (Not) | 0.015 | 0.059 | 0.264 | 0.79 | -0.1 | 0.13 |

*Table S1* Multilevel regression analyses in Experiments 1,2,4
